# Genetic polymorphisms of the Growth Hormone (GH) gene in Damascus and Black Bengal male goats

**DOI:** 10.1007/s11250-024-04253-y

**Published:** 2025-01-06

**Authors:** Mahmoud A. Moawad, Hadeer M. Aboshady, Mamdouh S. Abd-Alla, Nasser Ghanem, Ahmed Y. Abdel-Moneim, Masahide Nishibori, Takahiro Yonezawa, Hideyuki Mannen, Rania Agamy

**Affiliations:** 1 Animal Production Department, Faculty of Agriculture, Cairo University, Giza, 12613 Egypt; 2 Graduate School of Integrated Sciences for Life, Hiroshima University, Higashi-Hiroshima, 739-8528 Japan; 3 Graduate School of Agricultural Science, Kobe University, Kobe, Japan

**Keywords:** Single nucleotide polymorphism, Carcass cuts, Genetic marker, GH gene

## Abstract

Sustainable agriculture approaches necessitate a concerted effort from researchers to establish paths that meet global population needs without compromising environmental resources. Goats are unique among ruminants because of their ability to adapt to some of the harshest environments around the world. Growth Hormone (GH) gene is a major regulator of muscle mass growth. Therefore, our study aims to analyze the whole sequence of GH gene in order to identify various single nucleotide polymorphisms (SNPs) in Damascus and Black Bengal goats (BBG) and to predict the effect of mutant residues on the protein’s structure and function. Additionally, this study examined the effects of these SNPs on growth characteristics in Damascus goats. The study was designed to test 22 male goats. To obtain high-quality amplification of the GH gene, we utilized the nested PCR technique and performed paired-end sequencing for each fragment. Sequences alignment in the GH gene of Damascus and BBG goats unveiled 25 SNPs with different frequencies within the two breeds. Seven SNPs identified in coding regions comprised four synonymous variants (719 C → T, 731 G → A, 1610 C → T and 1625 C → T) and three non-synonymous variants (1112 G → A, 1459 C → T and 1470 C → T). The SNP 1112 G → A led to the substitution of Glutamic acid with Lysine (E/K) at amino acid position 137. The SNP 1459 C → T led to the substitution of Alanine with Valine (A/V) at amino acid position 160. Meanwhile, the SNP 1470 C → T led to the substitution of Leucine with Phenylalanine(L/F) at amino acid position 164. The SNP 1112 G → A was predicted to have a deleterious effect on the protein’s structure and function. The SNPs 914 G → A and 1112 G → A showed significant differences (P < 0.05) between genotypes in pre-slaughter weight and almost carcass cuts in Damascus goats, indicating their possible use in breeding programs as a potential genetic marker for weight/size in goats as well as in assessing and choosing members of this breed for meat production.

## Introduction

Agriculture faces a great challenge due to the increasing global population, which requires increasing food production while implementing effective and sustainable production techniques. The ability of ruminants, especially small ruminants, to use low-value inputs and produce high-value outputs is well known. Among ruminants, goats have distinction for their resilience in surviving in some of the world’s most inhospitable environments, which has led to the moniker "the poor man's cow." This emphasizes how important they are to small-scale farming systems. The world goat population is over one billion; around 90% this total number is found in developing countries (The Mediterranean basin, Asia, and Africa) (FAO Statistics 2020).

The goat (*Capra hircus*) was one of the first animals to be domesticated, more than 10,000 years ago (Nomura et al. 2013). Furthermore, it has a high diversity of behaviours, physiological and genetic characteristics, which enabled it to display exceptional adaptability in a wide range of ecosystems. From an economic point of view, they are raised worldwide as a vital source of milk, meat, and fiber.

In Egypt, there are five native goat breeds including Baladi, Barky, Zaraibi, Sinawi, and Saidi (Latif et al. 1987; El-Sayed et al. 2017). These breeds are distributed in the Upper Egypt, Nile Delta and the desert rangelands particularly around the northwestern coast, according to the availability of different pastures. Damascus goat, commonly known as the Shami, is a Syrian native breed, one of the most popular breeds used in genetic improvement schemes to increase the productivity of native Egyptian goats (El-Sayed et al. 2017). The Damascus goat has a reddish-brown coat color. The hair, ears and head are long with a Roman nose. The adult female has an average live body weight of 65 ± 5 kg and 75 ± 5 kg for the male (Mavrogenis et al. 2006). The breed is considered as one of the best dual-purpose breeds of the Middle East under semi-intensive or intensive production systems, with medium to high fertility (80% to 90%) (Jnied et al. 2013). Meanwhile, the Black Bengal Goat (BBG) is considered the native breed of Bangladesh and one of Bangladesh's most influential small ruminants that produce red meat (Hossain 2021). The BBG is also registered as one of the indigenous breeds of goat in India (National Bureau of Animal Genetic Resources, India 2012). The BBG is of small body size, weighs 25–30 kg as an adult, has small horns, small legs, and a slim body anatomy structure (Hossain 2021). Both breeds differ in body size, growth rate, and body weight.

Chevon, the meat produced from goats, has increased in global consumption compared to other red meats because of its favourable nutritional and biological properties. These include a high protein content (up to 29%) (Stajic and Pisinov 2021), low level of cholesterol and saturated fatty acids, a high content of favourable unsaturated fatty acids, and it is a vital source of minerals, including iron and potassium as well as vitamin B12 (Mazhangara et al. 2019). Goat meat production is a significant sector within Egypt's animal production, serving as a key source of red meat. Unfortunately, the lack of feed ingredients and availability of pasture led to a decrease in the goat population from 3,973,692 to 1,243,646 during the period 2017 to 2022, subsequently decreased the contribution of goat meat to the red meat supply and increased chevon price (Ministry of Agriculture and Land Reclamation 2022). Goats are primarily bred randomly in developing countries, with few targeted selection programs. Identifying the genetic factors that control phenotypic variation of growth traits in goats could accelerate genetic improvement. Moreover, identifying genetic variants underlying growth performance would support efforts to increase production.

Growth hormone (GH) is the main growth regulator in animals. GH is released from the anterior lobe of the pituitary gland, which is necessary for various biological processes, including growth, metabolism, lactation, and reproduction (Machlin 1976). GH improves nitrogen uptake and amino acid absorption into tissue proteins, which leads to an increase in muscle mass. Additionally, it improves the ability of ribosomes to translate messenger RNA, enhances RNA and DNA polymerase synthesis and promotes cell division into different tissues (Mahrous et al. 2018). The Caprine GH has been mapped on chromosome 19. It is encoded by the goat GH gene ID (100861230) with 1634 base pairs (bp) and consists of five exons divided by four introns (National Center for Biotechnology Information, NCBI (n.d.)).

Several studies have been conducted to investigate the GH gene polymorphism and its effect on milk production (Lan et al. 2007; Dettori et al. 2013), growth rate (An et al. 2011, 2010; Buranakarl et al. 2024; Hua et al. 2009; Pandya et al. 2021; Sarmah et al. 2020), and body measurements (An et al. 2010; Hua et al. 2009). Investigations of genetic variability in the goat GH gene have highlighted significant correlations with body weights in the Indian Sirohi breed (Kumar et al. 2011). Detailed studies have been conducted on the polymorphism of GH genes, which play a crucial role in growth characteristics. For example, Ayele et al. (2024) identified two alleles (A and B) and two genotypes in the GH gene using the PCR–RFLP technique for Saanen, Alpine, and Boer goat breeds. They reported an association between these polymorphisms and chest width in Saanen and Alpine goats, as well as a link between body weight and heart girth in Boer goats. Similarly, in Egyptian Awassi lambs, PCR–RFLP analysis identified three genotypes and two alleles in the GH gene. The presence of allele A was associated with consumed feed, body mass index and post-weaning daily growth, while allele B was related to marketing weight and loin and tail percentages (El-Mansy et al. 2023). Additionally, Kumar et al. (2024) reported two genotypes in GH gene using the PCR–RFLP technique and these genotypes were found to have significant effect on annual greasy fleece weight in Harnali sheep. Several other studies have examined variances in the GH gene using RFLP or PCR-SSCP and reported a significant impact of the identified polymorphisms on growth rates and body characteristics in animals (Gitanjli et al. 2020; Sarmah et al. 2020; Pandya et al. 2021; Saputra et al. 2021; Ouchar et al. 2023). However, few publications have studied polymorphisms across the entire GH gene (El-halawany et al. 2019).

There is a lack of information regarding the genetic variability of the GH gene in Damascus and BBG goats. Thus, limits the effective use of genetic variation in animal breeding programs. To our knowledge, no studies have been conducted to detect the polymorphic types or SNPs across the entire GH gene in Damascus and BBG goats, or to explore the relationship between genetic variations and growth characteristics in the Damascus goat breed. Therefore, the objectives of this study were first to identify and compare polymorphisms in the entire GH gene between Damascus (medium to large body size breed) and BBG goats (small body size breed) and to predict the effect of mutant residues on the protein's structure and function. Secondly, assess whether there might be a relationship between the discovered polymorphisms and growth traits in the Damascus goat breed raised in Egypt, which could have potential applications in breeding programs.

## Materials and methods

### Experimental animals and blood samples

Damascus goat has been raised at the Agricultural Experimental Station of Cairo University, which is located in Giza, Egypt (30°02′ N and 31°13′E, with an altitude of 30 m). Fourteen Damascus kids were raised from 4 months until 12 months as a group in semi-shaded open yards. The goats were fed according to the NRC requirements (2007) on concentrate mixture. The daily supplementation starts with 250 g of concentrates per head at the beginning of experiment and ended with 500 g per head at the end of study. The concentrate mixture comprised of 50% yellow corn, 25%wheat bran, 15% soybean cake, 5% sunflower seed cake 1.5% limestone, 1% common slate, 0.5%minerals, 0.2% vitamins mixture, 0.3% yeast extracted and 1.5% sodium bicarbonate. The crude protein and TDN were estimated as 16% and 70%, respectively. Animals were fed ad libitum on Darawa (*Zea maize L.*) and/or Egyptian clover (*Trifolium alexandrinum*) according to its availability. Mineral blocks and water were easily available during the day. During the raising period, body weight was measured monthly. Blood was collected from the jugular veins of the 14 Damascus healthy animals and received in a 3 ml vacutainer tube coated with EDTA as an anticoagulant, then preserved under −20 °C until the DNA was extracted. Then, at 12 months age the animals were slaughtered and fabricated into primal cuts to determine the carcass composition. Immediately prior to slaughter, the body weight was recorded for all animals. They were then slaughtered after a fasting period of 18 h at the Agricultural Experiments Station, Faculty of Agriculture, Cairo University. The kids were slaughtered in accordance with Muslim (halal) tradition, where the major blood vessels in the nech and the throat and major blood vessels in the neck were severed at the atlanto-occipital joint. Following bleeding, the carcass was skinned, opened and internal organs were taken out. Afterwards, the hot carcass weight (about 1 h after slaughtering) was recorded. Subsequently, the carcass was divided into roughly two equal halves longitudinally. The left and right halves of the carcass were cooled for 24 h at 4 °C. Each chilled carcass half was weighed. Then, each carcass's chilled half was divided into eight sections in accordance with Abdel-Moneim (2009). The eight carcass parts that were identified included: neck, middle neck (from the 1st to the 5th dorsal vertebra), and best end of neck (from the 6th to the 13th dorsal vertebra), shoulder, brisket, lion, flank and leg.

### DNA extraction

Genomic DNA was isolated from whole blood samples of Damascus goats using Thermo Scientific GeneJet Whole blood genomic DNA purification kit (Thermo Fisher Scientific, USA), following the manufacturer's guidelines Thermo Fisher Science. The concentration and purity of DNA were assessed using a Nanodrop spectrophotometer at optical densities of 260 and 280 nm. Moreover, for comparison study was done using eight Black Bengal Goat (BBG). DNA samples of this breed were provided from the institution of Graduate School of Agricultural Science, Kobe University, Japan. According to the Cairo University -Hiroshima University co-supervision program of master's students AY2023, all complementary studies following DNA extraction were conducted at Hiroshima University, Japan.

### Primers and polymerase chain reaction

**Fig. 1:**
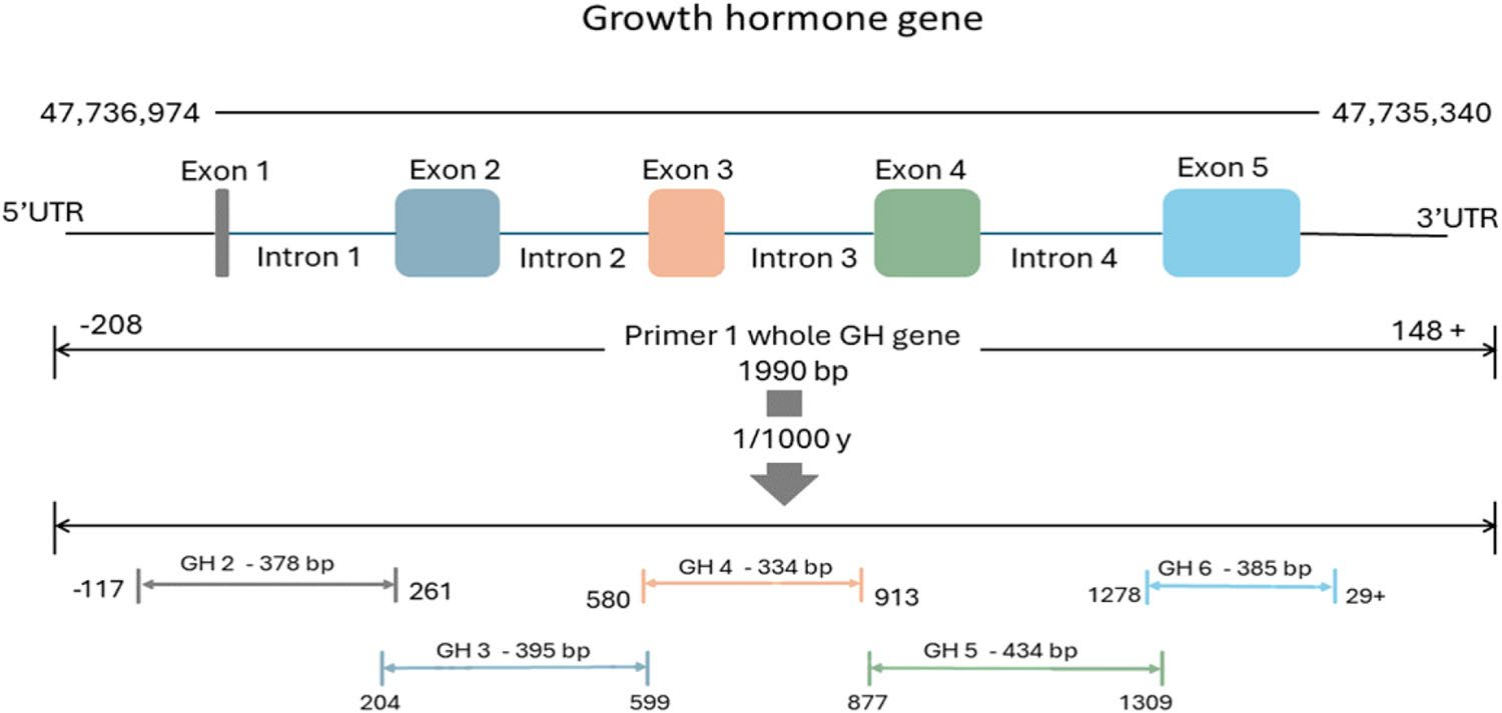
Growth hormone gene and amplified parts design

Polymerase chain reaction (PCR) was used to amplify whole GH gene (GH1) and five tested GH parts (GH2 to GH6) using nested PCR technique (Green and Sambrook 2019). The Primers used to amplify, GH1 and GH6 were designed by primer3plus, while other primers for GH2, GH3, GH4 and GH5 were reported previously (Wickramaratne et al. 2010; El-Halawany et al. 2019) (Table 1 and Fig. 1).

**Table 1:** The primers information

Part	Primer name	primer Sequence	Annealing temperature	Reference	PCR product size
Whole GH gene	GH1 F	ACGGGAACAGGATGAGTGAGAG	58 C°	designed by primer3plus	1990 bp
	GH 1 R	CTGACCCCACCCCCTAGAATAG			
Part 1	GH 2 F	GGGGGAAAGGGAGAGAGAAG	61 C°	(Wickramaratne et al. 2010; El-Halawany et al. 2019)	378 bp
	GH 2 R	CCCTAGGGAGAGACCAGGAG			
Part 2	GH 3 F	GATCAGGCATCCAGCTCTCT	61 C°		395 bp
	GH 3R	TCACTGCCTTATTCGGAACC			
Part 3	GH 4 F	GGTTCCGAATAAGGCAGTGA	68 C°		334 bp
	GH 4 R	CACCACCACCAACCATCAT			
Part 4	GH 5 F	GTTGGATGGCAGTGGAGGATG	65 C°		434 bp
	GH 5 R	AAGAGGTTTCTCCTGGGTCAAGA			
Part 5	GH 6 F	AGCAGCCCAGTCTTGACCCAGGA	65 C°	designed by primer3plus	385 bp
	GH 6 R	GGAGGGGTAACAACAGATGGCTG

The polymerase chain reaction was performed using KOD FX Neo DNA polymerase (Toyobo, Osaka, Japan). The PCR components were 20 µl, due to the recommendation of the company TOYOBO, contained genomic DNA 80 ng /20 µl for only amplified GH1; after that we used PCR product GH1 as a templet for other reactions 1 µl from diluted 1:1000, 0.6 µl each primer with concentration 10 pmol /µl, 10 µl KOD FX PCR buffer, 4 µl 2 mM dNTPs, 0.4 µl KOD FX polymerase and up to a total volume with PCR grade water. The PCR thermal cycling program consisted of an initial pre-denaturation step at 94 °C for 2 min, followed by 30 cycles of denaturation at 98 °C for 10 s, annealing at a specific temperature for 30 s depending on the annealing temperature of primers used and extension depending on fragment length (GH1 was 1 min and other primers were 20 s) at 68 °C, final cooling at 4 °C / ∞. The PCR products were detected by gel electrophoresis for 35 min with agarose gel 1.5% for GH1 and 2% for others. Then, the gels were soaked in ethidium bromide for 15 min and checked out with an ultraviolet transilluminator.

For PCR product purification GH parts 1, 2,3 and 5 were purified by enzymes Exonuclease I (EXO1) 0.75 µl + Shrimp Alkaline Phosphatase 0.75 µl for 15 µl of PCR product, then incubated in PCR at 37 °C / 15 min followed by 80 °C / 15 min. GH parts 4 was purified using a FastGene Gel/PCR Extraction Kit (Nippon Genetics, FG-91302, Tokyo, Japan).

### GH amplified sequence, and polymorphisms detection

The five amplified PCR fragments that comprise the GH gene were sequenced in paired-end reading by the FASMAC Corporation Sequencing service (Kanagawa, Japan). After gene sequencing, the contig between the forward and revers sequences was analyzed by GeneStudio software (GeneStudio TM Professional Edition 2011). We aligned the sequence against NCBI (National Center for Biotechnology Information (NCBI)) goat GH gene ID (100861230) using MEGA 11 software (Tamura et al. 2021). Whereas, novoSNP software (Weckx et al. 2005) was used to identify single nucleotide polymorphisms (SNPs) within each breed and among the reference sequences of the two goat breeds. The Ensembl Variant Effect Predictor (VEP) (McLaren et al. 2016) website was utilized to predict the SNP variant's effect on the GH gene. The prediction of the detrimental effect of non-synonymous (missense) variances in amino acids was studied by the SIFT (sorting intolerant from tolerant) website (Sim et al. 2012).

### Prediction of protein structure

AlphaFold Colab2 (Jumper et al. 2021) website was utilized to predict the growth hormone protein's three-dimensional structure (3D). Furthermore, a comparison between the changed amino acid in the protein structure predicted and the reference protein 3D structure from the uniport website GH1 (P67931) was conducted using ICM pro (Molsoft L.L.C. (n.d.)).

### Statistical analysis

The influence of different SNPs on the living body weight and carcass cuts of Damascus kids were analyzed through one-way ANOVA and Duncan's Multiple Range tests using Origin (pro), version 2024 (OriginLab Corporation).

## Results

### Detection and frequencies of SNPs

A total of twenty-five SNPs were detected when the GH gene was aligned against the reference NCBI gene sequence for each of the two goat breeds (ID: 100861230) (Table 2).

**Table 2:** Genotype and allele frequency of identified SNPs in growth hormone gene in Damascus and BBG goats

SNP	Position	Chro.19 position	Genotypes	Damascus Allele frequency		BBG Allele frequency	
				Ref. allele	SNP allele	Ref. allele	SNP allele
65 A → G	Intron 1	47,736,907	AA, GG	A (0.64)	G (0.36)	A (0.38)	G (0.62)
67 A → G	Intron 1	47,736,905	AA, GG	A (0.64)	G (0.36)	A (0.38)	G (0.62)
68 A → C	Intron 1	47,736,904	AA, CC	A (0.64)	C (0.36)	A (0.38)	C (0.62)
70 Insertion C	Intron 1	47,736,902	Not, Ins C	- (0.64)	C (0.36)	- (0.38)	C (0.62)
133 G → A	Intron 1	47,736,841	GG, AA, AG	G (0.82)	A (0.18)	G (0.44)	A (0.56)
626 C → T	Intron 2	47,736,348	CC, TT, TC	C (0.54)	T (0.46)	C (0.38)	T (0.62)
632 G → A	Intron 2	47,736,341	GG, AG	G (1.00)	ND	G (0.94)	A (0.06)
719 C → T	Exon 3	47,736,256	CC, TT, TC	C (0.50)	T (0.50)	C (0.31)	T (0.69)
731 G → A	Exon 3	47,736,244	GG, AA, AG	G (0.86)	A (0.14)	G (1.00)	ND
794 C → T	Intron 3	47,736,181	CC, TT, TC	C (0.79)	T (0.21)	C (0.25)	T (0.75)
810 T → C	Intron 3	47,736,163	TT, CC, CT	T (0.86)	C (0.14)	T (1.00)	ND
914 G → A	Intron 3	47,736,059	GG, AA	G (0.50)	A (0.50)	G (0.75)	A (0.25)
957 G → A	Intron 3	47,736,016	GG, AG	G (0.89)	A (0.11)	G (0.69)	A (0.31)
965 G → A	Intron 3	47,736,008	GG, AG	G (0.96)	A (0.04)	G (0.94)	A (0.06)
1112 G → A	Exon 4	47,735,861	GG, AG	G (0.86)	A (0.14)	G (0.56)	A (0.44)
1174 G → A	Intron 4	47,735,799	GG, AA, AG	ND	A (1.00)	G (0.62)	A (0.38)
1178 C → G	Intron 4	47,735,795	CC, GC	C (0.96)	G (0.04)	C (1.00)	ND
1258 G → T	Intron 4	47,735,715	GG, TG	G (1.00)	ND	G (0.94)	T (0.06)
1332 C → T	Intron 4	47,735,641	CC, TC	C (0.82)	T (0.18)	C (0.75)	T (0.25)
1349 A → G	Intron 4	47,735,624	AA, GG, GA	A (0.50)	G (0.50)	A (0.25)	G (0.75)
1418 C → T	Intron 4	47,735,555	CC, TC	C (0.79)	T (0.21)	C (1.00)	ND
1459 C → T	Exon 5	47,735,514	CC, TC	C (0.93)	T (0.07)	C (1.00)	ND
1470 C → T	Exon 5	47,735,503	CC, TC	C (0.96)	T (0.04)	C (1.00)	ND
1610 C → T	Exon 5	47,735,363	CC, TC	C (0.71)	T (0.29)	C (1.00)	ND
1625 C → T	Exon 5	47,735,348	CC, TC	C (0.96)	T (0.04)	C (1.00)	ND

*ND* not detected

**Fig. 2:**
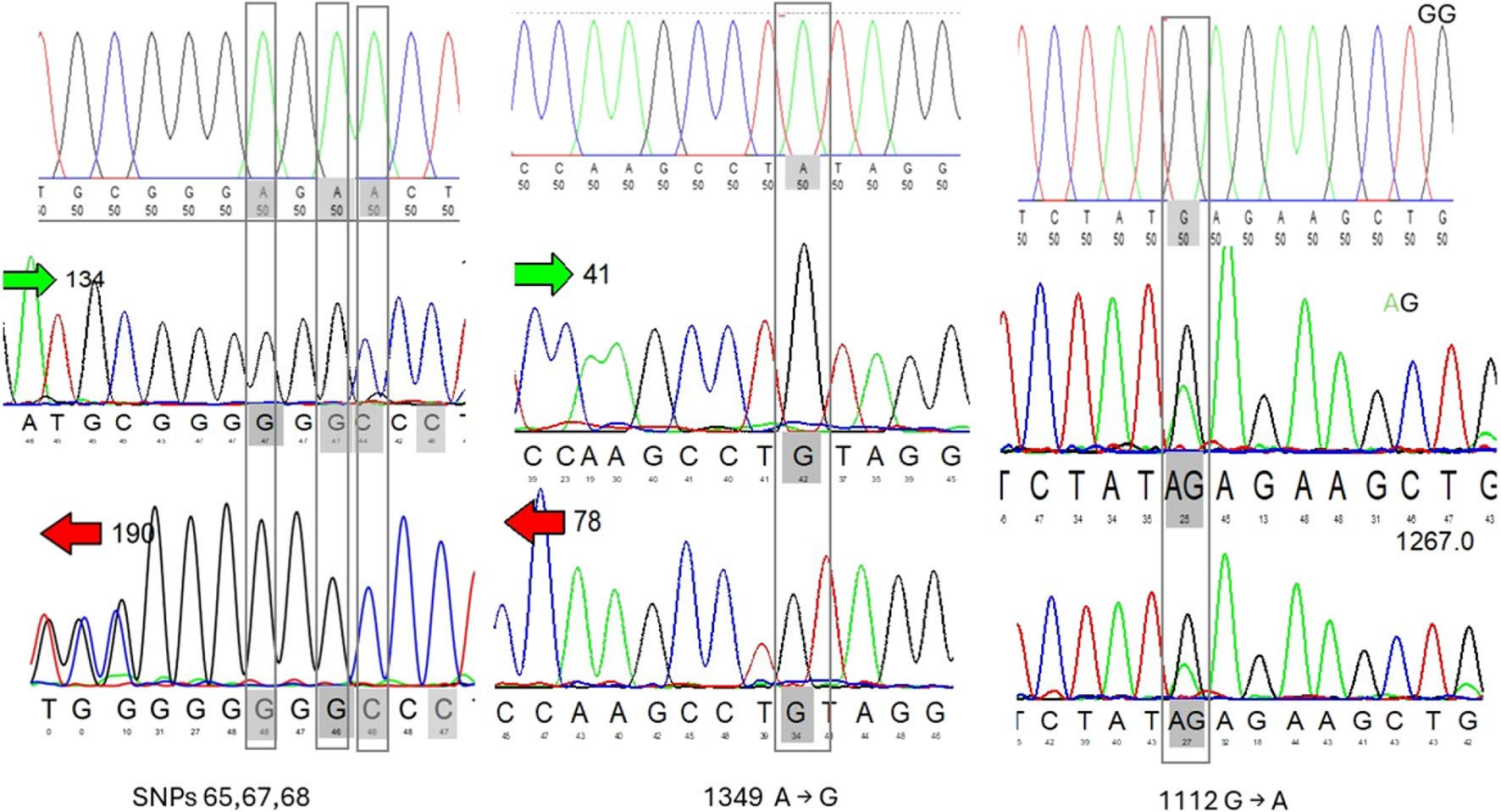
Analyzing the chromatogram to align sequences and showed potential single nucleotide polymorphisms (SNPs) and genotypes

**Fig. 3:**
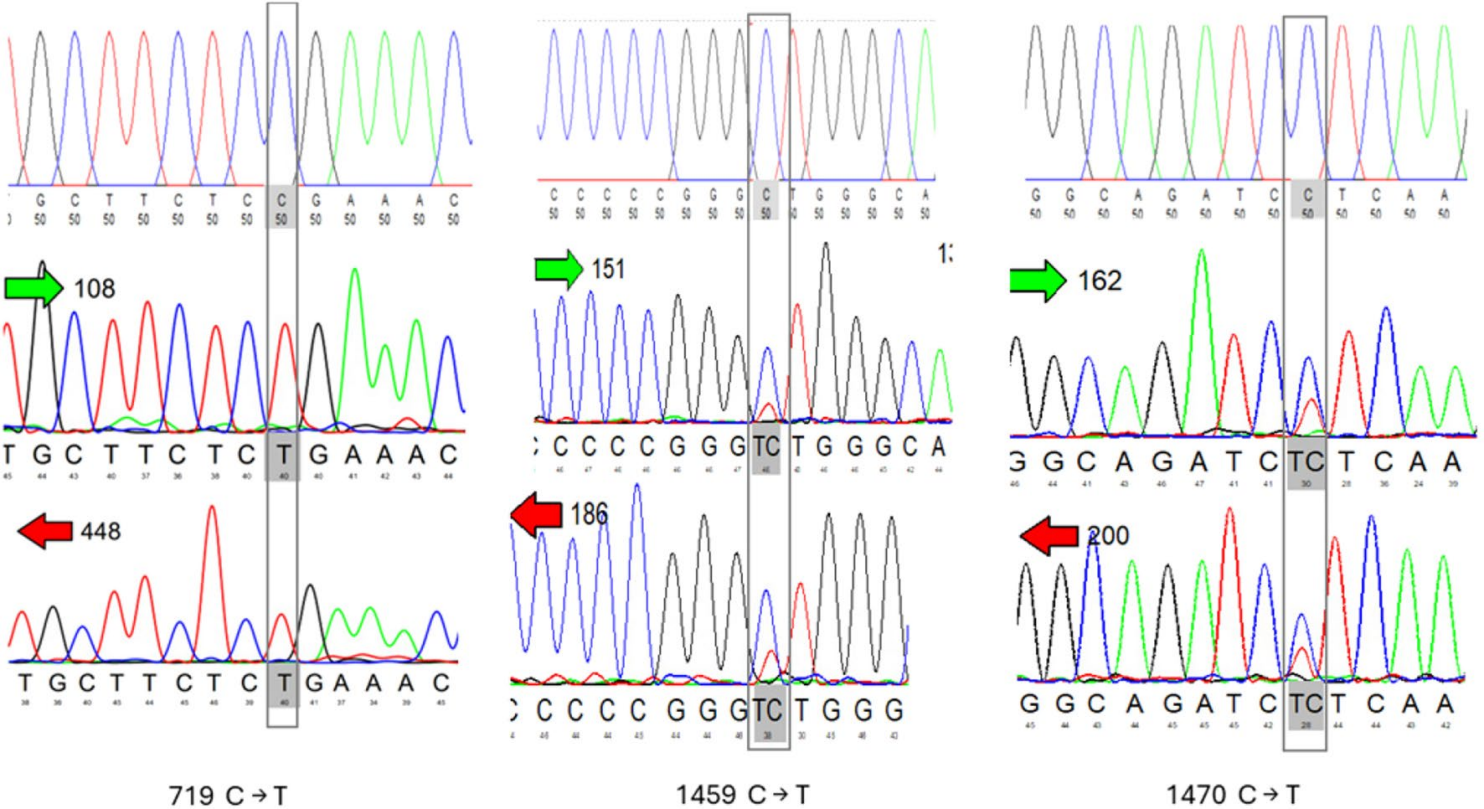
Analyzing the chromatogram to align sequences and identify potential single nucleotide polymorphisms (SNPs) in exons parts

The majority of these SNPs were detected in non-coding regions (introns) representing 72% of the total SNPs identified. Four SNPs were found in intron 1 (65 A → G, 67 A → G, 68 A → C, and 70 INC C). These four SNPs were observed to appear simultaneously due to the proximity of their bases. Consequently, this pattern was considered as a single genotype (Fig. 2). The SNP 1349 A → G in intron 4 was notably detected in all Damascus and BBG individuals, showing dominance for the GA and AA genotypes. Variations within coding regions (exons) constituted 28% of the total SNPs identified. These SNPs were distributed across exon 3 (719 C → T, 731 G → A), exon 4 (1112 G → A), and exon 5 (1459 C → T, 1470 C → T, 1610 C → T, 1625 C → T) (Fig. 3).

Table 2 displays the allele frequencies of different SNPs in the Damascus and BBG goat breeds. The comparison detected 23 SNPs within Damascus goats, while two unique SNPs (1258 G → T and 632 G → A) were detected exclusively in the BBG breed. On the other hand, the BBG breed exhibited 17 SNPs, with an additional 8 unique SNPs (731 G → A, 810 T → C, 1178 C → G, 1418 C → T, 1459 C → T, 1470 C → T, 1610 C → T and 1626 C → T) detected only in Damascus goats.

A comparison of the data highlights notable variations in allele frequencies between the two groups, indicating unique genetic differences specific to each breed. The allele frequencies of SNP 65 A → G, SNP 67 A → G, and SNP 68 A → C in intron 1 were contrasted in both breeds. Damascus goats showed a consistent pattern where the reference alleles were more frequent, whereas the alternative alleles were more frequent in BBG goats. The allele frequency for SNP 1112 G → A exhibited notable differences between breeds, with a high frequency in BBG individuals compared to Damascus individuals. The SNP 1174 G → A in intron 4 exhibited complete polymorphism to allele A in Damascus individuals, unlike BBG individuals, who had a higher frequency of allele G, suggesting region-specific genetic variations or evolutionary adaptations. The alternative alleles in BBG goats in SNPs 133 G → A, 626 C → T, 719 C → T, 794 C → T and 1349 A → G exhibited a greater frequency compared to the reference alleles. In contrast, the Damascus goat reference alleles had higher or equal frequencies compared to the alternatives. On the other hand, SNP 914 G → A showed a higher frequency of the reference allele than the alternative in BBG goats. Both breeds shared the same trend in the frequency of alleles at SNPs 957 G → A, 965 G → A, 1112 G → A and 1332 C → T, with the reference alleles being more prevalent than the alternative alleles.

### Synonymous and non-synonymous (missense) SNPs and amino acid mutations

Seven Single Nucleotide Polymorphisms (SNPs) were identified within coding regions of the GH gene in both breeds (Table 3). Four SNPs were predicted to be synonymous variants in positions 719, 731, 1610, and 1625. These alterations maintain the same amino acid sequence, often reflecting the redundancy of the genetic code. Meanwhile, three SNPs were predicted to be non-synonymous variants. Non-synonymous variants, which alter the amino acid sequence, often have the potential to impact various levels of protein structure, protein helices, physiological function, and ultimately the phenotypes of animals. For example, positions 1112, 1459, and 1470 lead to the substitution of Glutamic acid with Lysine (E/K) at amino acid position 137, Alanine with Valine (A/V) at position 160, and Leucine with Phenylalanine (L/F) at position 164. These changes in the encoded protein may affect the biochemical properties of the growth hormone and potentially influence physiological processes.

**Table 3:** A comprehensive description of Single Nucleotide Polymorphisms (SNPs) that were identified within coding regions of the GH Gene

SNP	Position	mRNA position	Codon	Amino Acid position	Mutation type
719 C → T	Exon 3	243	TCC - TCT	81 - Serine	synonymous variant
731 G → A	Exon 3	255	CCG - CCA	85 - Proline	synonymous variant
1112 G → A	Exon 4	409	GAG - AAG	137 - Glutamic to Lysine (E/K)	Non-synonymous variant
1459 C → T	Exon 5	479	GCT - GTT	160 - Alanine to Valine (A/V)	Non-synonymous variant
1470 C → T	Exon 5	490	CTC - TTC	164 - Leucine to Phenylalanine (L/F)	Non-synonymous variant
1610 C → T	Exon 5	630	TTC - TTT	210 - Phenylalanine	synonymous variant
1625 C → T	Exon 5	645	TGC - TGT	216 - Cysteine	synonymous variant

Sorting intolerant from tolerant (SIFT) was used to predict the effect of non-synonymous (missense) variants on protein function. SIFT is a web-based tool that relies on an algorithm to predict the deleterious effect of amino acid substitutions at specific positions in a protein. The SIFT scores range from 0 to 1, with scores classified as deleterious from 0.00 to 0.10, borderline from 0.101 to 0.20, and tolerant from 0.201 to 1.00. The SNP 1112 has a SIFT score of 0.05, while SNP 1459 and SNP 1470 have scores of 0.11 and 0.47, respectively. Overall, based on these SIFT scores, the SNP 1112 was predicted to have a deleterious effect, indicating functional significance, while SNPs 1459 and 1470 were predicted to have less impact on protein functions.

### Prediction of GH protein three-dimensional model

**Fig. 4:**
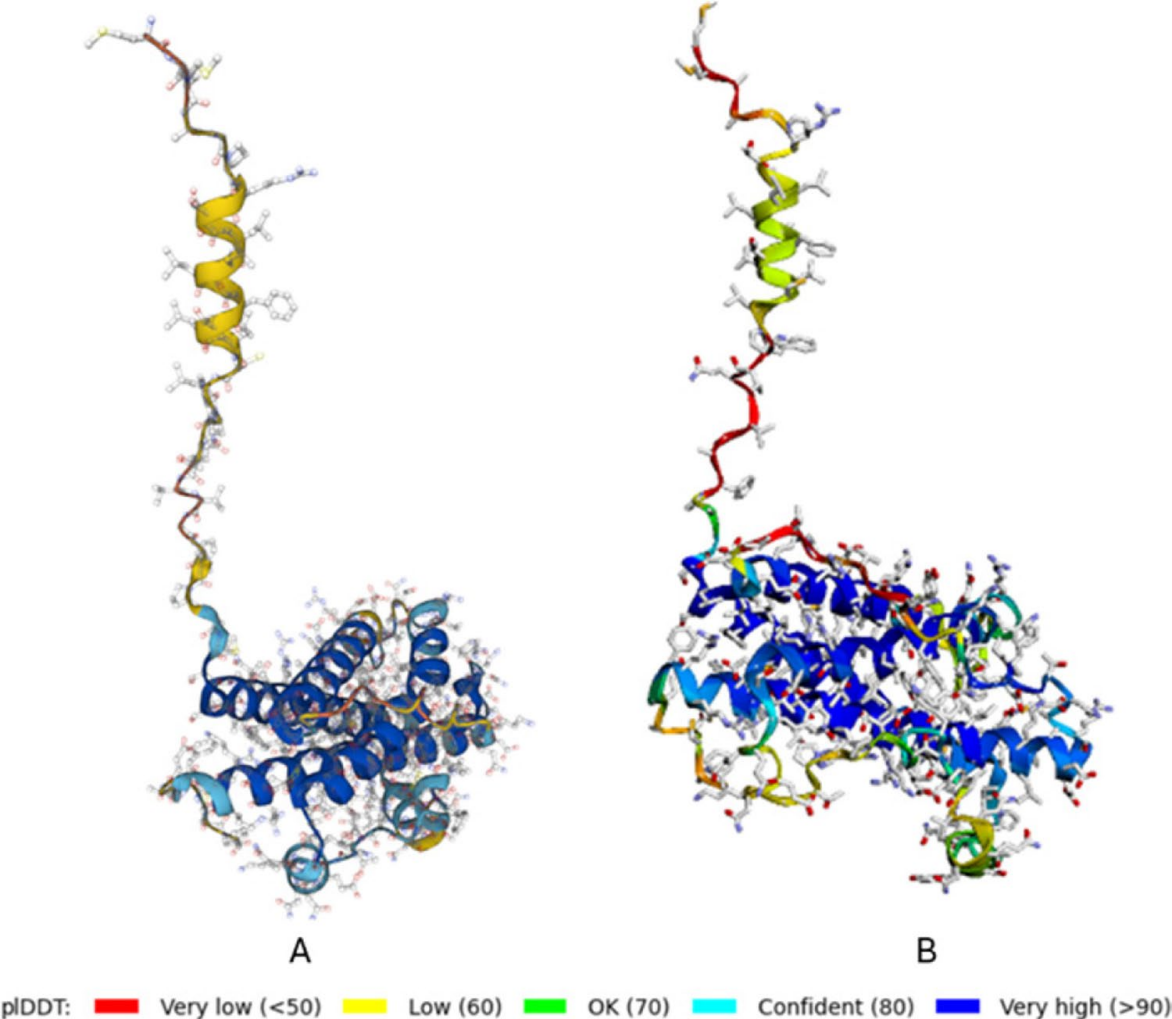
Comparing 3-D model of the GH protein (**A**) with 3-D model of the GH protein containing three SNPs (**B**)

The three-dimensional model of the growth hormone protein was folded using AlphaFold Colab 2. The structure, composed of 217 amino acids, was compared to the UniProt GH1 (P67931) structure, as illustrated in Fig. 4. The initial 26 amino acids at the N-terminus represent a signal peptide necessary for hormone secretion. This peptide is cleaved during secretion process, producing the mature form of GH, which consists of 191 amino acids. The mature protein includes two disulfide bridges and four alpha helices arranged in a distinctive anti-parallel manner. One of the three SNPs detected as missense (1112 G/A) is located in the third helix. The other two SNPs (1459 C/T and 1470 C/T) are located in the disulfide chain.

The substitution of SNP 1112 results in the change from glutamic acid, a negatively charged and acidic amino acid, to lysine, a positively charged and basic amino acid. Conversely, the SNP 1459 substitution from alanine to valine does not cause a notable change, as both amino acids are hydrophobic and nonpolar aliphatic R group. For SNP 1470, both leucine and phenylalanine are hydrophobic and nonpolar. However, leucine has an aliphatic R group, while phenylalanine has an aromatic R group.

**Fig. 5:**
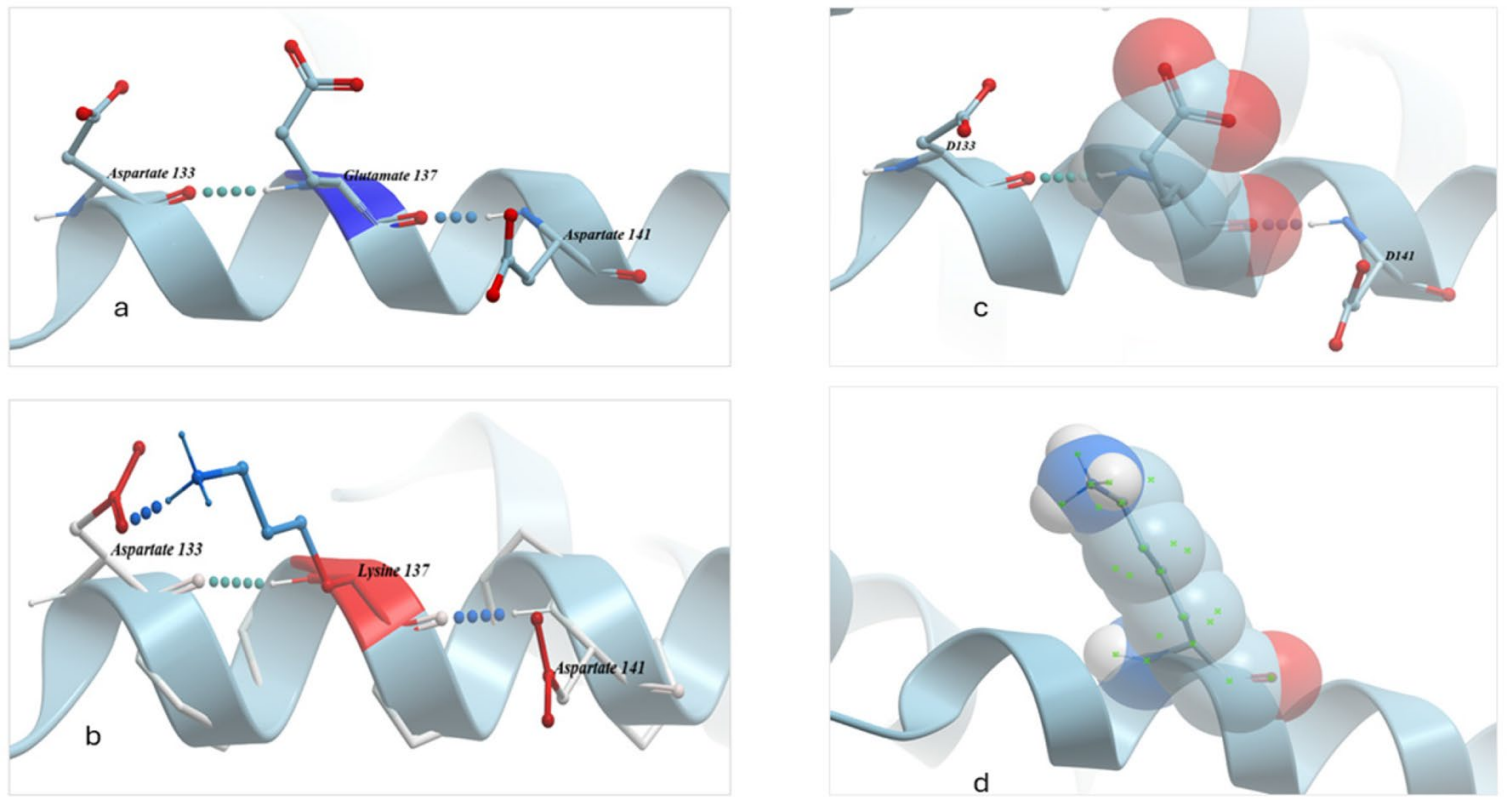
A comparison of chemical structure, bonds molecules connection in position 137 in case of Glutamic acid (**a**) and substitute with lysine (**b**) and the size of space-filling physical molecule for glutamic acid (**c**) and lysine(**d**)

The ICM pro software was used to compare the reference residues and SNP mutant residues within the protein's architecture and to analyze the intermolecular interactions between molecules. The substitution of SNP 1112 G → A resulted in the change of glutamic acid to lysine (Fig. 5) at position 137, located on the surface of the third helix loop. The Chemical structures, bonds between amino acid molecules which regulate folding mechanisms and electron density maps showed differences due to this SNP. The results revealed that while the chemical structures of glutamic acid and lysine are somewhat similar, lysine is distinguished by a long aliphatic side chain containing a positively charged amino group (NH3 +), which allows it to interact with other molecules. During protein helix folding, glutamic acid forms one bond each with Aspartic acid 133 and Aspartic acid 141. The substitution to lysine is predicted to result in a new bond forming with Aspartic acid 133, leading to two bonds with this molecule. The electron density map predicted changes due to the substitution of glutamic acid with lysine, which occupies a larger physical space.

**Fig. 6:**
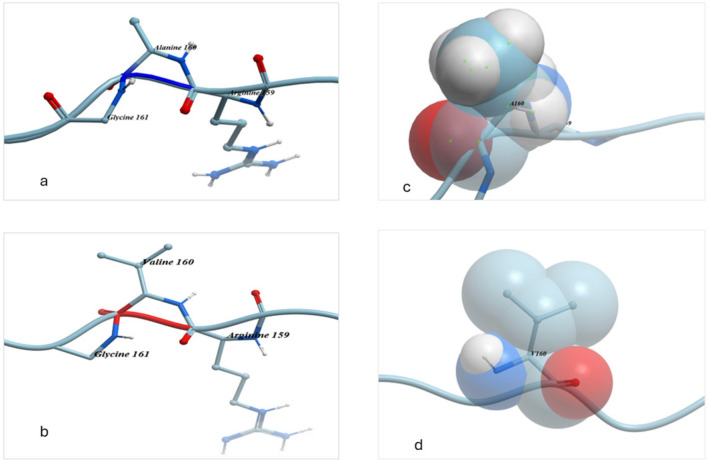
A comparison chemical structure, bonds molecules connection in position 160 in case of alanine (**a**) and substitute with valine (**b**) and the size of space-filling physical molecule for alanine (**c**) and valine (**d**)

The substitution of SNP 1459 C → T resulted in the change of alanine to valine (Fig. 6) at position 160, located on the disulfide chain. The results revealed that both alanine and valine are hydrophobic and have non-polar aliphatic R group. However, valine has a longer side chain containing two methyl groups, which gives it a higher hydrophobicity level. The small side chain in alanine allows for greater flexibility during protein folding. In contrast, the longer chain in valine has an increased capacity to interact with other non-polar amino acids. The space-filling physical structure of valine is slightly larger than that of alanine.

**Fig. 7:**
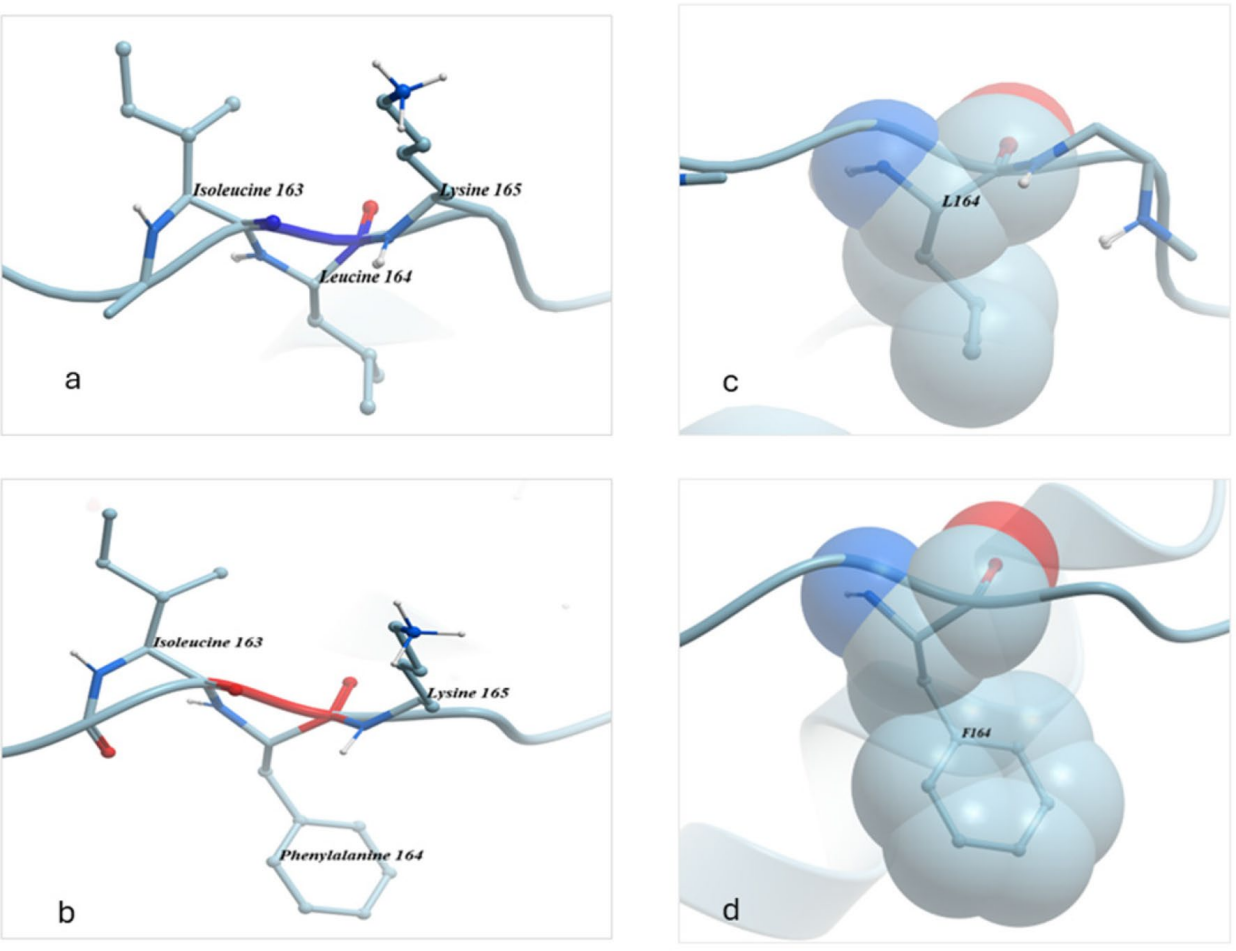
A comparison chemical structure, bonds molecules connection in position 164 in case of leucine (**a**) and substitute with phenylalanine (**b**) and the size of space-filling physical molecule for leucine (**c**) and phenylalanine (**d**)

The substitution of SNP 1470 C → T resulted in the change of leucine to phenylalanine (Fig. 7) at position 164, located on the disulfide chain. The results revealed that while both leucine and phenylalanine are hydrophobic, leucine is nonpolar with an aliphatic chain containing a methyl group, whereas phenylalanine is aromatic with a benzene ring. Leucine’s aliphatic R group contributes to greater stability during protein folding. Phenylalanine, with its benzene ring, can participate in pi-stacking interactions and hydrophobic interactions with other aromatic residues within proteins, potentially affecting the folding and stability of the protein. The space-filling physical structure of phenylalanine is notably larger than that of leucine, which may influence the surface shape of the protein.

### The impact of SNPs on animal weight and carcass traits

The effects of SNPs on pre-slaughter weight (PSW) and carcass traits in Damascus goats are presented in Table 4. The results showed that these SNPs may influence the animals’ body weight and carcass cuts (weight of hot carcass, leg, loin, flank, shoulder, brisket, neck, best end of neck and Middle neck). The results indicated that several SNPs exhibited significant differences between genotypes in body weight and different carcass cuts. SNP 914 G → A with GG genotype was associated with higher performance in almost all carcass traits, except for leg weight, which did not show significant differences in the analysis of variance. Notably, the genotype AA at SNP 914 was associated with low performance in the majority of tested traits. The results for SNP 1112 G → A, which was found to have a deleterious effect on hormone structure, showed that animals with the AG genotype had significantly lower performance than with the GG genotype in almost all traits. The analysis of variance showed significantly lower weights (P < 0.05) for the AG genotype at SNP 1112 in hot carcass, loin, neck, pre-slaughter, leg, shoulder, brisket, best end of neck and middle neck. Meanwhile, the flank weight did not show a significant difference.

**Table 4:** Effects of Single Nucleotide Polymorphisms (SNPs) on pre-slaughter weight (PSW) and carcass traits in Damascus goats

		PSW (Kg)	HCW (Kg)	LEW (Kg)	LOW (Kg)	FLW (Kg)	SHW (Kg)	BRW (Kg)	NEW (Kg)	BEON (Kg)	MN (Kg)
		$\overline{X }\pm SE$	$\overline{X }\pm SE$	$\overline{X }\pm SE$	$\overline{X }\pm SE$	$\overline{X }\pm SE$	$\overline{X }\pm SE$	$\overline{X }\pm SE$	$\;\overline{\overline X\pm SE}$	$\;\overline{\overline X\pm SE}$	$\overline{X }\pm SE$
65 −70	AA	28.1 ± 1.98	13.8 ± 1.3	2.0 ± 0.16	0.53 ± 0.08	0.14 ± 0.04	1.5 ± 0.13	0.45 ^**b**^ ± 0.05	0.77 ± 0.06	0.65 ± 0.07	0.49 ± 0.07
	GG	29.9 ± 0.66	14.6 ± 0.29	2.1 ± 0.05	0.59 ± 0.05	0.12 ± 0.01	1.5 ± 0.05	0.65 ^**a**^ ± 0.04	0.79 ± 0.03	0.68 ± 0.02	0.42 ± 0.01
133 G/A	GG	28.0 ± 1.75	13.8 ± 1.13	2.0 ± 0.14	0.55 ± 0.07	0.14 ± 0.03	1.5 ± 0.11	0.47 ± 0.05	0.76 ± 0.06	0.65 ± 0.06	0.49 ± 0.06
	AG	30.5 ± 0.5	14.5 ± 0.33	2.1 ± 0.06	0.54 ± 0.06	0.14 ± 0.01	1.5 ± 0.07	0.65 ± 0.06	0.78 ± 0. 02	0.71 ± 0.02	0.42 ± 0.02
	AA	30.5 ± 0.00	15.5 ± 0.00	2.2 ± 0.00	0.66 ± 0.00	0.09 ± 0.00	1.6 ± 0.00	0.70 ± 0.00	0.89 ± 0.00	0.66 ± 0.00	0.42 ± 0.00
626 C/T	CC	26.4 ± 2.1	12.9 ± 1.5	1.9 ± 0.19	0.49 ± 0.10	0.11 ± 0.04	1.4 ± 0.14	0.40 ^**b**^ ± 0.04	0.72 ± 0.07	0.59 ± 0.08	0.45 ± 0.08
	TC	33.0 ± 3.0	16.5 ± 2.1	2.3 ± 0.25	0.65 ± 0.04	0.24 ± 0.10	1.7 ± 0.22	0.60 ^**a**^ ± 0.05	0.92 ± 0.08	0.81 ± 0.12	0.64 ± 0.08
	TT	29.9 ± 0.66	14.6 ± 0.29	2.1 ± 0.05	0.59 ± 0.05	0.12 ± 0.01	1.5 ± 0.05	0.65 ^**a**^ ± 0.04	0.79 ± 0.03	0.68 ± 0.02	0.42 ± 0.01
719 C/T	TT	29.9 ± 0.54	14.6 ± 0.24	2.1 ± 0.04	0.59 ± 0.04	0.12 ^b^ ± 0.01	1.5 ± 0.04	0.64 ^**a**^ ± 0.03	0.80 ± 0.03	0.69 ± 0.01	0.45 ± 0.03
	CC	26.4 ± 2.1	12.9 ± 1.5	1.9 ± 0.19	0.49 ± 0.10	0.11 ^**b**^ ± 0.04	1.4 ± 0.14	0.40 ^**b**^ ± 0.04	0.72 ± 0.07	0.59 ± 0.08	0.45 ± 0.08
	TC	36.0 ± 0.00	18.5 ± 0.00	2.5 ± 0.00	0.69 ± 0.00	0.33 ^**a**^ ± 0.00	1.9 ± 0.00	0.64 ^**a**^ ± 0.00	0.99 ± 0.00	0.93 ± 0.00	0.72 ± 0.00
731 G/A	GG	28.7 ± 1.6	14.0 ± 1.0	2.1 ± 0.13	0.53 ± 0.06	0.14 ± 0.03	1.5 ± 0.10	0.50 ± 0.05	0.77 ± 0.05	0.65 ± 0.06	0.46 ± 0.06
	AA	30 ± 0.00	14.4 ± 0.00	2.0 ± 0.00	0.61 ± 0.00	0.14 ± 0.00	1.5 ± 0.00	0.55 ± 0.00	0.84 ± 0.00	0.70 ± 0.00	0.57 ± 0.00
	AG	28.5 ± 1.0	14.4 ± 0.3	2.0 ± 0.05	0.63 ± 0.05	0.13 ± 0.02	1.4 ± 0.01	0.66 ± 0.03	0.76 ± 0.04	0.69 ± 0.05	0.45 ± 0.01
794 C/T	CC	28.1 ± 2.0	13.8 ± 1.3	2.0 ± 0.16	0.53 ± 0.08	0.14 ± 0.04	1.5 ± 0.13	0.45 ^**b**^ ± 0.05	0.77 ± 0.06	0.65 ± 0.07	0.50 ± 0.07
	TT	30.5 ± 0.00	15.5 ± 0.00	2.2 ± 0.00	0.66 ± 0.00	0.09 ± 0.00	1.6 ± 0.00	0.70 ^**ab**^ ± 0.00	0.89 ± 0.00	0.66 ± 0.00	0.42 ± 0.00
	TC	29.8 ± 0.83	14.4 ± 0.26	2.1 ± 0.06	0.57 ± 0.05	0.13 ± 0.01	1.5 ± 0.05	0.64 ^**a**^ ± 0.04	0.77 ± 0.02	0.69 ± 0.02	0.43 ± 0.01
914 G/A	GG	31.0 ^**a**^ ± 1.1	15.8 ^**a**^ ± 0.62	2.2 ± 0.08	0.64 ^**a**^ ± 0.05	0.18 ^**a**^ ± 0.03	1.6 ^**a**^ ± 0.08	0.60 ^**a**^ ± 0.03	0.87 ^**a**^ ± 0.03	0.74 ^**a**^ ± 0.04	0.55 ^**a**^ ± 0.04
	AA	26.2 ^**b**^ ± 2.0	12.2 ^**b**^ ± 1.1	1.9 ± 0.16	0.45 ^**b**^ ± 0.07	0.08 ^**b**^ ± 0.02	1.3 ^**b**^ ± 0.11	0.44 ^**b**^ ± 0.07	0.67 ^**b**^ ± 0.05	0.56 ^**b**^ ± 0.06	0.38 ^**b**^ ± 0.07
1112 G/A	GG	30.2 ^**a**^ ± 0.91	15.2 ^**a**^ ± 0.56	2.2 ^**a**^ ± 0.07	0.62 ^**a**^ ± 0.04	0.15 ± 0.03	1.6 ^**a**^ ± 0.07	0.58 ^**a**^ ± 0.04	0.83 ^**a**^ ± 0.03	0.71 ^**a**^ ± 0.03	0.52 ^**a**^ ± 0.04
	AG	24.0 ^**b**^ ± 3.5	10.6 ^**b**^ ± 1.8	1.7 ^**b**^ ± 0.25	0.32 ^**b**^ ± 0.05	0.07 ± 0.04	1.2 ^**b**^ ± 0.17	0.37 ^**b**^ ± 0.08	0.60 ^**b**^ ± 0.09	0.49 ^**b**^ ± 0.12	0.30 ^**b**^ ± 0.06
1332 C/T	CC	29.4 ± 1.1	14.6 ± 0.63	2.1 ± 0.07	0.57 ± 0.06	0.14 ± 0.02	1.5 ± 0.06	0.57 ± 0.05	0.81 ± 0.03	0.68 ± 0.03	0.47 ± 0.04
	TC	27.4 ± 3.4	13.1 ± 2.2	1.9 ± 0.29	0.52 ± 0.10	0.13 ± 0.07	1.4 ± 0.22	0.45 ± 0.07	0.71 ± 0.11	0.62 ± 0.13	0.47 ± 0.13
1418 C/T	CC	29.3 ± 1.3	14.4 ± 0.70	2.1 ± 0.08	0.59 ± 0.06	0.13 ± 0.02	1.5 ± 0.07	0.57 ± 0.05	0.80 ± 0.03	0.67 ± 0.04	0.45 ± 0.04
	TC	27.9 ± 2.7	13.6 ± 1.8	2.0 ± 0.24	0.50 ± 0.08	0.14 ± 0.05	1.4 ± 0.18	0.47 ± 0.06	0.74 ± 0.10	0.64 ± 0.10	0.49 ± 0.10
1459 C/T	CC	29.2 ± 1.3	14.5 ± 0.85	2.1 ± 0.11	0.59 ± 0.05	0.14 ± 0.03	1.5 ± 0.09	0.55 ± 0.04	0.79 ± 0.05	0.67 ± 0.05	0.49 ± 0.05
	TC	26.3 ± 4.8	12.2 ± 1.8	1.9 ± 0.18	0.36 ± 0.07	0.09 ± 0.05	1.3 ± 0.14	0.39 ± 0.13	0.68 ± 0.05	0.59 ± 0.13	0.35 ± 0.07
1610 C/T	CC	28 ± 2.7	13.6 ± 1.8	2.0 ± 0.23	0.55 ± 0.08	0.12 ± 0.05	1.4 ± 0.18	0.50 ± 0.07	0.75 ± 0.10	0.62 ± 0.10	0.46 ± 0.10
	TC	29.3 ± 1.3	14.5 ± 0.71	2.1 ± 0.08	0.56 ± 0.7	0.14 ± 0.02	1.5 ± 0.07	0.55 ± 0.05	0.79 ± 0.03	0.68 ± 0.04	0.47 ± 0.04

*PSW* pre-slaughter weight (Kg), *HCW* hot carcass weight (Kg), *LEW* leg weight (Kg), *LOW* loin weight (Kg), *FLW* flank weight (Kg), *SHW* shoulder weight (Kg), *BRW* brisket weight (Kg), *NEW* neck weight (Kg), *BEON* best end of neck weight (Kg), *MN* middle neck weight (Kg). Trait mean for different SNP genotype that do not has the same letter differ significantly from each other (*P* < 0.05).$\overline{X }$: mean, SE: stander error

Additionally, some SNPs showed a remarkable effect on specific parts of the carcass. The SNPs (65 A → G, 67 A → G, 68 A → C, and 70 INC C) together as one genotype, 626 C → T, SNP 719 C → T and 794 C → T showed significant effect on brisket weight with favorable effect for the SNP allele. Besides, the SNP 719 C → T had an observed effect on flank weight.

The SNPs 810 T → C, 957 G → A, 965 G → A, 1174 G → A, 1178 C → G, 1470 C → T and 1625 C → T, were detected in a limited number of animals, as well as SNP 1349 A → G was found in almost animals with genotype AG. Therefore, it was not feasible to study the statistical effects of these SNPs on animal weight and carcass traits. Additionally, the limited number of animals caused in reduced reliability of these SNPs effect on the growth traits. Therefore, the study of SNPs effects in a larger sample of animals is imperative to consider as selection markers.

## Discussion

The candidate gene approach represents a potential strategy for investigating the association between specific genes and traits, providing valuable insights for enhancing breeding programs through marker-assisted selection. The growth hormone (GH) gene is one of the key genes responsible for regulating the expression of growth characteristics in both mammalian and non-mammalian species. Various studies on GH polymorphisms have been conducted in humans (Esteban et al. 2007), rabbits (Safaa et al. 2023), ducks (Wu et al. 2012), Nile tilapia fish (Jaser et al. 2017), beef cattle (Sedykh et al. 2020), sheep (Esen and Elmacı 2022) and goats (Buranakarl et al. 2024). Therefore, the main objective of the current research work was to identify single nucleotide polymorphisms (SNPs) in the entire GH gene of both Damascus and Black Bengal goats and to predict the effect of mutant residues on the protein's structure and function. Additionally, the study aimed to assess the relationship between the discovered SNPs and growth traits in the Damascus goat breed raised in Egypt.

Goat growth performance is influenced by various non-genetic factors at different levels, including management practices, nutrition, and health status. Characteristics such as breed, litter size, and sex significantly affect growth performance (Alade et al. 2008). Environmental conditions, including season of birth, temperature, humidity, and food availability, have also been reported to significantly influence goat growth traits (Mandal et al. 2010). Additionally, growth is influenced by the management system (intensive or extensive), breeding practices, housing type, and herd management strategies (Tena 2020). In our study, we aimed to minimize the impact of non-genetic factors on growth by selecting males with minimal time between births and raising them under similar environmental conditions as the group. This controlled approach facilitates a more accurate evaluation for the relationship between the discovered SNPs and growth traits in the Damascus goat by reducing the effects of non-genetic variables.

Studying the GH gene sequences in Damascus and BBG breeds revealed 25 single nucleotide polymorphisms (SNPs), including three that are considered non-synonymous (nsSNPs). Unfortunately, there is incomplete knowledge about the variations in the GH gene in Damascus goats and BBG, as limited research has been conducted to detect some parts of the GH gene in both breeds. In the current study, intron 1 exhibited a hotspot region, which was identified as a genotype previously reported in Damascus goats (Abdelhafez et al. 2022). Meanwhile, the fifth SNP (133 G → A) was reported as a marker for Zaraibi goat (El-Halawany et al. 2019). In Exon 3, two SNPs were detected in our study, both of them (SNP 719 C → T and 731 G → A), were previously documented in three Egyptian goat breeds (Baladi, Barki and Zaraibi), as well as in Sarda dairy goats (Dettori et al. 2013; El-Halawany et al. 2017, 2019). The SNPs 810 T → C and 1174 G → A were previously discovered in introns 3 and 4, respectively, in the three Egyptian goat breeds (El-Halawany et al. 2019). The SNPs 1332 C → T and 1349 A → G in intron 4 were detected in both Osmanabadi and Sangamneri goats and were associated with an effect on average daily gain for the first SNP (Wickramaratne et al. 2010). However, in the current study, it did not show any effect on body weight or carcass cuts. Additionally, within the same fourth intron, the SNP 1418 C → T was detected in Egyptian Baladi goats (El-Halawany et al. 2019). In exon 5, the SNP 1459 C → T was identified in turkey lambs (Esen and Elmacı 2022), while SNP 1610 C → T was detected in Egyptian goat breeds (Baladi, Barki and Zaraibi) and Sarada goats (Dettori et al. 2013; El-Halawany et al. 2019). The SNP 1625 C → T was also detected in Sarada and Sangamneri goats (Wickramaratne et al. 2010; Dettori et al. 2013). Based on our knowledge and a review of previous research, 10 SNPs were identified as novel SNP. Of these ten SNPs, two were detected in coding regions, 1112 G → A and 1470 C → T in exon 4 and 5, respectively. While in the non-coding regions, the following novel SNPs were detected: in intron 2, 626 C → T and 632 G → A; in intron 3, 794 C → T, 914 G → A, 957 G → A, 965 G → A and in intron 4, 1178 C → G and 1258 G → T. The SNPs 632 and 1258 are specific to BBG, whereas SNPs 1178 and 1470 are unique to the Damascene goat breed. In contrast, research conducted by Abdelhafez et al. (2022) and Mahrous et al. (2018) identified three SNPs in intron 1, eight gaps at the end of the exon 3 fragment and one SNP in exon 3 of the GH gene in Damascus goats, which were not observed in our study.

Similarly, in BBG goats a SNP in exon 2 was detected and associated with growth traits (Buranakarl et al. 2024). Additionally, variable genotypes were identified in exons 4 and 5, including six and eight SNPs, respectively (Gupta et al. 2007). However, none of these SNPs was observed in our study. The SNPs in intron 1 showed a similar frequency in our study compared to the study by Abdelhafez et al. (2022), where the alternative allele frequency was 0.36 instead of 0.2. On the other hand, studies on BBG goat found five genotypes AA, CC, AB, AC and AD, with variations in allele frequencies, supporting the hypothesis of multiple SNPs in this region (Dayal et al. 2014, 2016). These genotypes were also found to have a significant effect on birth weight, with the highest values in animals with the AC genotype and the lowest values in animals with the CC genotype (Dayal et al. 2014, 2016).

The novel missense SNP (non-synonymous) 1112 G → A in exon 4 was identified in both breeds, with a higher frequency of the A allele observed in BBG goats (0.44) compared to 0.14 in Damascus goats, highlighting a notable difference between small and large goat breeds. Furthermore, the significantly negative effect of this substitution on pre-slaughter weight and the majority of carcass cuts in Damascus goats confirms the impact of this SNP on the goat’s weight and size. In this context, a study of the GH gene exon 4 in Boer goats reported two genotypes, CC and CD, with a high frequency of the C allele being 0.93. The CD genotype was associated with lower performance in body measurements and weights (Hua et al. 2009). However, these differences were not statistically significant (P > 0.05). The SIFT score for this SNP was 0.05, indicating that it is predicted to have a deleterious effect on protein function. Additionally, the substitution of glutamic acid with lysine in position 137 (resulting from this SNP) is located on the surface of the third helix loop. According to a study in humans (Brooks and Waters 2010), the third helix is crucial for the connection with the growth hormone receptor at site 2. Growth hormone initially binds to a single monomeric growth hormone receptor on the cell surface and subsequently induces receptor dimerization by recruiting a second growth hormone receptor through interaction at site 2. The study of the three-dimensional structure revealed notable differences at all levels, including the structure of the substituted amino acid, the bonds formed between molecules during protein folding and the space filling by physical molecules. These differences collectively indicate notable changes in protein structure due to this SNP, which may enhance the deleterious effects of the 1112 SNP. In contrast, the study of exon 4 in BBG identified seven SNPs, comprising two synonymous SNPs and five non-synonymous SNPs (Gupta et al. 2007). None of these SNPs were detected in our study.

The missense SNP 1459 C → T in exon 5 was identified exclusively in the Damascus goat. This SNP was also detected in Turkish lambs (Esen and Elmacı 2022) while studying genetic variability in exon 5. Lambs with heterozygotes showed significant effects on shorter body length, thicker carcass backfat and higher weight in the neck, shoulder, and leg regions (Esen and Elmacı 2022). In contrast, our study found no significant effect of this SNP on body weight and carcass characteristics. However, heterozygote individuals exhibited lower values for all measured traits, with no significant effect, which could be attributed to a combination of low allele frequency and a small sample size. The SIFT score for 1459 SNP is 0.11, indicating a borderline effect on protein structure and functions. The substitution of alanine to valine at position 160 (resulting from SNP 1459 C → T) is located on the disulfide chain and is generally tolerated due to the position of the variant. In humans, the description of structure, functional and reference molecule modeling study have reported that disulfide bridges are not necessary for the biological activity of the molecule, both chain variants of growth hormone (GH) can exhibit full biological activity (Sami and Amtul 2007). The structure of the substituted amino acid, the bonds between molecules and the space filling by physical molecules do not show notable difference due to the SNP, although the longer chain in valine, with two methyl groups, has more capacity to interact with other non-polar amino acids.

The novel missense SNP (non-synonymous) 1470 C → T, identified in exon 5, is exclusive to the Damascus goat. Unfortunately, the low frequency of this SNP precludes the ability to test its effect on body weight or carcass traits. However, the SIFT score for this SNP is 0.47, indicating a minimal predicted effect on protein structure and function. Contrasting the study of the 3-D structure with the substitution leucine to phenylalanine in position 164, located on the disulfide chain, revealed a notable change in the substituted amino acid structure. These changes affected protein stability during protein folding and altered the space-filling properties of the physical molecule, potentially leading to a change in the surface shape of the protein. The previous study of exon 5 in BBG did not identify this SNP (Gupta et al. 2007). Nevertheless, they identified eight SNP that were not detected in our study. Several studies (An et al. 2010; El-Halawany et al. 2019; Gupta et al. 2007; Lan et al. 2007; Rashijane et al. 2022; Wickramaratne et al. 2010) have identified SNPs in exon 5 and their association with growth traits. In Chinese goats, two SNP were identified in exon 5 by An et al. (2010), which significantly affect body weight, withers height, body length and chest girth. Wickramaratne et al. (2010) reported SNP 2055 C/T in the Osmanabadi breed, which was also found in our study at position 1625 in exon 5, but they reported no significant effect of this SNP on growth parameters. In the Boer goats, (Rashijane et al. 2022) identified a SNP in exon 5 which significantly affected body weight, with the heterozygotes genotype showing high performance.

Single Nucleotide Polymorphisms (SNPs) in the GH gene contribute to genetic variability among individuals. In the current study, specific SNPs, such as 914 G → A and 1112 G → A, have been statistically linked to growth traits. These identified SNPs assist breeders in selecting individuals with desirable genetic traits and excluding those with undesirable traits at an early age, thereby accelerating the breeding process. Our study revealed 25 SNPs, of which 10 were novel discoveries. Furthermore, eleven SNPs were found to align with those previously reported in other Egyptian breeds by El-Halawany et al. (2019, 2017), providing evidence of potential shared ancestry and a common breeding history among Egyptian goat breeds. Additionally, three SNPs were detected that align with those reported in Indian breeds by Wickramaratne et al. (2010), and one SNP was consistent with those found in Turkish lambs by Esen and Elmacı (2022).

Several studies have demonstrated a significant statistical association between polymorphisms in the GH gene and growth traits. For instance, SNPs 1332 C → T and 1459 C → T, reported by Esen and Elmacı (2022) and Wickramaratne et al. (2010), significantly influence various growth parameters, including average daily gain, neck weight, shoulder weight, leg weight, carcass backfat, and body length. Additionally, El-Mansy et al. (2023) identified polymorphisms in exons 2 and 3 that significantly affected loin percentage, tail percentage, neck percentage, body mass index, and post-weaning daily gain in Egyptian Awassi lambs. Similarly, Ayele et al. (2024) reported an association between polymorphisms in exons 2 and 3 and chest width, body weight, and heart girth in Saanen, Alpine, and Boer goats. These findings highlight the consistency of polymorphism patterns across various studies, emphasizing the significant role of genetic polymorphisms as selection markers for growth and morphological traits in different goat breeds. This consistency not only reinforces the relevance of these markers in genetic research but also demonstrates their importance in breeding programs aimed at enhancing and improving growth traits.

Several publications have studied variations in the GH gene using RFLP or PCR-SSCP without detecting our reported SNPs (Alakilli et al. 2012; Singh et al. 2015; Ilham et al. 2016; Susilorini et al. 2017; Bayan et al. 2018; Gooki et al. 2018, 2019; Gitanjli et al. 2020; Sarmah et al. 2020; Pandya et al. 2021; Saputra et al. 2021; Ouchar et al. 2023). These studies reported a significant correlation and impact of genetic polymorphisms on growth traits and body characteristics of animals. Finnaly, the importance of investigating genetic variations in the growth hormone gene has been confirmed, highlighting the necessity for further research to confirm genetic polymorphisms that could be used in genetic selection depending on their relationship with growth traits.

## Conclusion

In the present study, the entire growth hormone gene was sequenced in Damascus and Black Bengal goat breeds. Sequence analysis revealed 25 SNPs in total; 23 SNPs in Damascus goats and only 17 SNPs in BBG. Seven of them were located in coding region, three of which were non- synonymous and lead to amino acid changes. The presence of non- synonymous SNPs led to a notable alteration in the 3D structure of the GH gene. One SNP in exon 4 was predicted to have a deleterious effect, which was also associated with low production performances (weigh and carcass cuts) in Damascus goats. Further investigation with a larger sample size is required to emphasis these relationships before implement in marker-assisted selection.

## Data Availability

Additional details concerning the results and methodologies will be provided by the author upon request for correspondence.
